# Coculture with hematopoietic stem cells protects cardiomyocytes against apoptosis via paracrine activation of AKT

**DOI:** 10.1186/1479-5876-10-115

**Published:** 2012-06-06

**Authors:** Mark Rosenberg, Matthias Lutz, Constantin Kühl, Rainer Will, Volker Eckstein, Jutta Krebs, Hugo A Katus, Norbert Frey

**Affiliations:** 1 Department of Internal Medicine III (Cardiology and Angiology), University Medical Center Schleswig-Holstein, Campus Kiel, Schittenhelmstr.12, D-24105, Kiel, Germany; 2 Department of Internal Medicine III, University of Heidelberg, Heidelberg, Germany; 3 Department of Internal Medicine V, University of Heidelberg, Heidelberg, Germany

**Keywords:** Stem cells, Cardiomyocytes, Apoptosis, Paracrine

## Abstract

**Background:**

Previous experimental studies concluded that stem cells (SC) may exert their beneficial effects on the ischemic heart by paracrine activation of antiapoptotic pathways. In order to identify potential cardioprotective mediators, we performed a systematic analysis of the differential gene expression of hematopoietic SC after coculture with cardiomyocytes (CM).

**Methods:**

After 48 h of coculture with neonatal rat ventricular CM (NRVCM), two consecutive cell sorting steps generated a highly purified population of conditioned murine hematopoietic SC (>99%). Next, a genome-wide microarray analysis of cocultured vs. monocultured hematopoietic SC derived from three independent experiments was performed. The analysis of differentially expressed genes was focused on products that are secretable and/or membrane-bound and potentially involved in antiapoptotic signalling.

**Results:**

We found CCL-12, Macrophage Inhibitory Factor, Fibronectin and connexin 40 significantly upregulated in our coculture model. An ELISA of cell culture supernatants was performed to confirm secretion of candidate genes and showed that coculture supernatants revealed markedly higher CCL-12 concentrations. Moreover, we stimulated NRVCM with concentrated coculture supernatants which resulted in a significant reduction of apoptosis compared to monoculture-derived supernatant. Mechanistically, NRVCMs stimulated with coculture supernatants showed a higher level of AKT-phosphorylation, consistent with enhanced antiapoptotic signaling.

**Conclusion:**

In summary, our results show that the interaction between hematopoietic SC and NRVCM led to a modified gene expression and induction of antiapoptotic pathways. These findings may thus at least in part explain the cardioprotective effects of hematopoietic SC.

## Background

Despite major advances in the treatment of coronary artery disease (CAD), acute myocardial infarction remains a major cause of death worldwide. In fact, the acute loss of blood supply potentially leads to apoptosis or necrosis of cardiomyocytes (CM) served by the infarct related artery resulting in ischemic cardiomyopathy and congestive heart failure. In the past the postmitotic heart had been considered a terminal differentiated organ unable to replace a significant loss of tissue such as that after an acute infarction [1]. This dogma has been challenged by the recent discovery of resident cardiac stem cells (SC) and the demonstration of hematopoietic SC that can home to the heart and transdifferentiate into cardiomyocytes [2,3]. These astonishing findings have led to the hypothesis that SC could be used for regeneration of infarcted myocardial tissue.

Therefore numerous studies have examined a potential therapeutic effect of bone marrow derived SC on myocardial function and regeneration after experimental myocardial infarction reviewed in [4]. Whereas some of these studies provided evidence for extensive myocardial regeneration after cellular cardiomyoplasty [5], others found no hematopoietic SC that had actually transdifferentiated into CM [6,7]. Yet, regardless of the variable effects on cardiac regeneration, virtually all of these studies found a significant improvement of cardiac function after cellular therapy. It is now generally accepted that SC therapy can favourably affect cardiac remodelling after myocardial infarction, although the scientific basis of this effect still remains to be elucidated.

Recent studies hypothesized that SC may exert their beneficial influence on cardiac repair by paracrine action on apoptosis or angiogenesis. It has been repeatedly shown that various SC types can produce and secrete a broad variety of cytokines, chemokines and growth factors that may be involved in cardiac repair [8-11].

On the basis of these experimental findings we postulated that the direct interaction of hematopoietic SCs with CM results in an upregulation of cardioprotective factors that can be secreted by hematopoietic SC and promote survival in cardiomyocytes. In order to identify interesting candidate genes, we performed a systematic analysis of the differential gene expression of hematopoietic SC after coculture with NRVCM with special emphasis on gene products involved in antiapoptotic signalling.

## Materials and methods

### Animals

All experiments were performed in accordance with the Guide for the Care and Use of Laboratory Animals published by US National Institutes of Health. The animal study protocols were revised and approved by the Institutional Committee for the Ethics of Animal Care and Treatment.

### Isolation and culture of neonatal rat ventricular cardiomyocytes (NRVCM)

1–2 days old Wistar rats (Charles River, Sulzfeld, Germany) were decapitized and hearts were harvested and minced in ADS. Subsequently, up to six digestion steps were carried out with pancreatin (Sigma, Munich, Germany, 0.6 mg/ml) and collagenase type II (Worthington, 0.5 mg/ml) in sterile ADS buffer containing 120 mmol/l NaCl, 20 mmol/l HEPES, 8 mmol/l NaH2PO4, 6 mmol/l glucose, 5 mmol/l KCl, 0.8 mmol/l MgSO4, pH 7.4. NRVCM were purified from contaminating fibroblasts using a Percoll (Amersham, Germany) gradient centrifugation step. Finally, NRVCMs were resuspended and cultured in Dulbecco’s modified Eagle’s medium (DMEM) containing 10% FCS, penicillin/streptomycin and L-glutamine (all from PAA, Linz, Austria) [12,13].

### Isolation, purification and labelling of hematopoietic SC

Isolation, purification and labelling of hematopoietic (lin-/c-kit+) SC were performed as described previously [14,15] with the magnetic-activated cell sorting (MACS)-kit from Miltenyi Biotech according to the manufacturer´s protocol. Briefly, C57Bl/6 mice were cervically dislocated. Shortly thereafter tibias and femurs were collected and flushed with phosphate-buffered solution (PBS) containing 2% FCS. To remove cell clumps, crude bone marrow was filtered through a 30 μm nylon mesh (Miltenyi Biotech). For the purpose of depleting mature blood cells such as T cells, B cells, monocytes/macrophages, granulocytes, erythrocytes as well as their commited precursors, bone marrow cells were incubated with a “cocktail” of biotinylated antibodies against a panel of “lineage” (lin) antigens, including CD5, CD45R (B220), CD11b, Anti-Gr-1 (Ly-6 G/C), 7–4 and Ter-119 (Lineage Cell Depletion Kit, Miltenyi Biotech). After addition of anti-biotin microbeads, lin-positive cells were separated using a magnetic column. Enrichment of the lin-negative cells for a subpool of cells expressing the SC marker c-kit/CD117 was performed by application of CD117 microbeads (Miltenyi Biotech). The purity of the separated cells was assessed by fluorescence-activated cell sorting (FACS) using a phycoerythrin labelled antibody against CD 117 (Pharmingen). Integrity and viability of purified lin-/c-kit + cells was confirmed by propidium iodide (PI) (Sigma) staining. In order to be able to detect and separate hematopoietic SC after culture with neonatal rat ventricular cardiac myocytes, lin-/c-kit + cells were labelled with the green fluorescent “cell tracker” carboxyfluorescein diacetate succinimydil ester (CFDA) (TefLabs). Lin-/c-kit + stem cells were therefore incubated for 30 min at 37°C with 5 μg/ml CFDA per 10^6^ cells. The staining process was concluded with two washing steps to assure clearance of any unbound CFDA.

### Coculture and separation of NRVCMs and Lin-/c-kit + stem cells

The main objective of this study was to analyze the influence of a direct interaction with NRVCM on the gene expression profile of hematopoietic SC. Therefore, a coculture system of NRVCM with hematopoietic SC was established as follows: NRVCMs were isolated from whole hearts of 1–2 days old Wistar rats, resuspended and cultured at a density of 2x10^6^ cells per well in an uncoated six well plate. After 48 h, hematopoietic SC were separated from whole bone marrow of C57Bl/6 mice and added at a density of 0.5x10^6^ cells per well resulting in a ratio of NRVCM to lin-/c-kit + stem cells of 4:1. Cell culture conditions remained unchanged at 37°C and 5% CO_2_ at all times. After 48 h the coculture was stopped and the supernatant removed, immediately frozen in liquid nitrogen and stored at −80°C until further analysis. Next, remaining cells were washed twice with PBS and dissolved using a Trypsin-EDTA solution (0.25% (w/v), Invitrogen/GIBCO). Conditioned SC and NRVCM were then separated by two consecutive cell sorting steps using a FACS-Vantage SE flow cytometry system running CellQuest software (BD). Reliable discrimination of hematopoietic SC and NRVCM was ensured utilizing both CFDA staining and different forward-scatter and side-scatter signals. Viability and integrity of the cells was confirmed by staining with propidium iodide (Sigma).

In order to be able to test the importance of direct cell-cell interaction between NRVCM and hematopoietic SC, conditioned cells were compared to identically treated lin-/c-kit + control cells that were held in a monoculture at a density of 2.5*10^6^ cells per well in uncoated six well plates. After 48 h, cells were washed twice with PBS and dissolved using a Trypsin-EDTA solution (0.25% (w/v), Invitrogen/GIBCO). Monocultured SC were also sorted twice with FACS before being used for subsequent experiments. Viability and integrity of monocultured cells was again confirmed by staining with propidium iodide (Sigma).

Further processing of conditioned SC, NRVCM and supernatants is described in more detail in the Additional file 1: Methods section. This includes the experimental procedures of RNA isolation and purification, ELISA assays, the detection of apoptotic NRVCM as well as the stimulation of NRVCM with concentrated conditioned media and immunoblotting. Since most of the study results refer to analysis of the genetic profile of hematopoietic SC, we decided to describe the process of microarray hybridization and microarray data analyses as well as real-time PCR analyses of gene expression in more detail in the upcoming paragraphs.

#### Probe labeling and Illumina sentrix BeadChip array hybridization

Biotin-labeled cRNA samples for hybridization on Illumina Mouse Sentrix-6 BeadChip arrays (Illumina, Inc.) were prepared according to Illumina’s recommended sample labeling procedure based on the modified Eberwine protocol [16]. In brief, 250 ng total RNA was used for complementary DNA (cDNA) synthesis, followed by an amplification/labeling step (in vitro transcription) to synthesize biotin-labeled cRNA according to the MessageAmp II aRNA Amplification kit (Ambion, Inc., Austin, TX). Biotin-16-UTP was purchased from Roche Applied Science, Penzberg, Germany. The cRNA was column purified according to TotalPrep RNA Amplification Kit, and eluted in 60 μl of water. cRNA Quality was checked using the RNA Nano Chip Assay on an Agilent 2100 Bioanalyzer and spectrophotometrically quantified (NanoDrop).

Hybridization was performed at 58°C in GEX-HCB buffer (Illumina Inc.) at a concentration of 50 ng cRNA/μl and unsealed in a wet chamber for 20 h. Spike-in controls for low, medium and highly abundant RNAs were added, as well as mismatch control and biotinylation control oligonucleotides. Microarrays were washed twice in E1BC buffer (Illumina) at room temperature for 5 min. After blocking for 5 min in 4 ml of 1% (wt/vol) Casein in phosphate buffered saline, Hammarsten grade (Pierce Biotechnology, Inc., Rockford, IL), array signals were developed by a 10-min incubation in 2 ml of 1 μg/ml Cy3-streptavidin (Amersham Biosciences, Buckinghamshire, UK) solution and 1% blocking solution. After a final wash in E1BC, the arrays were dried and scanned.

#### Scanning and data analysis

Microarray scanning was done using a Beadstation array scanner. Settings were adjusted to a scaling factor of 1 and PMT settings at 430. Data extraction was done for all beads individually, and outliers were removed when MAD (median absolute deviation) was >2.5. All remaining data points were used for the calculation of the mean average signal for a given probe, and the standard deviation for each probe was calculated. Array data were normalized using a quartile-normalization algorithm. To select differentially expressed trancripts, an empirical Bayes analysis was carried out. Transcripts were selected as differentially expressed with p-values <0.05 and corrected for multiple testing by a Benjamini & Hochberg algorithm [17]. Gene expression was visualized by scatterplots and heatmaps. Data analysis was performed with R (version 2.7.2) and Bioconductor (version 2.0.1) using the packages *beadarray* (version 1.8.0) and *limma* (version 2.14.7). Differentially expressed transcripts were further classified by cellular location of the corresponding proteins using the LOCATE subcellular localization database (http://locate.imb.uq.edu.au/) and according to their function using PANTHER Classification System (http://www.pantherdb.org/).

#### Quantitative real-time PCR analysis of gene expression

Real-time PCR (RT-PCR) was used to further validate data generated in the microarray analysis and performed as follows: 100 ng of DNase-digested total RNA of each condition was transcribed to first strand cDNA with the Transcriptor first strand cDNA synthesis kit (Roche, Germany). Reaction conditions were set as recommended by the supplier. Real time PCR primers (MWG Biotech) were designed assisted by the Primer 3 software (http://primer3.sourceforge.net/). Targets were normalized using oligonucleotide primers for GAPDH as an internal standard. The resulting amplicons contained an exon-intron-exon boundary. The ABI Prism 7700 Sequence Detection System (Perkin Elmer Applied Biosystems) and the Platinum SYBR Green qPCR SuperMix-UDG (Invitrogen) were used for performing real-time PCR from reverse transcribed cDNA samples following the manufacturer’s instructions. Specificity of the reactions was checked by melting curve analysis and by verifying the correct size of the product on a 2% agarose gel. Each PCR amplification was carried out in duplicate wells, using the following conditions: 2 min at 50°C, 2 min at 95°C, followed by a total of 40 temperature cycles (15 s at 95°C and 30 s at 60°C).

#### Statistical analysis

Data are presented as mean ± SEM. Statistical analysis was carried out with Students-*t*-Test, if not noted differently. *P* < 0.05 was considered statistically significant.

## Results

### Generation of a highly purified population of hematopoietic SC after coculture with NRVCM

Hematopoietic SC were isolated from male C57BL/6 mice by magnetic cell sorting, typically resulting in a yield of 1.1–1.4 x 10^8^ bone marrow cells per dissected mouse. After the separation process, approximately 0.9–1.2 x 10^6^ hematopoietic SC were obtained, corresponding to ~1% of all isolated bone marrow cells. FACS-analyses were performed to check for purity and viability of the separated stem cells. NRVCM were prepared from 1–2 days old Wistar Rats using a percoll gradient centrifugation.

**Figure 1:**
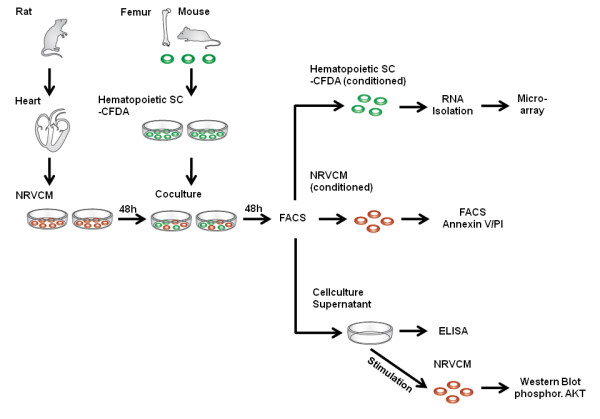
**shows the experimental setup.** After 48 h of coculture with NRVCM two cell sorting steps generated a highly purified population of conditioned murine hematopoietic SC that were used for RNA isolation and microarray analysis. Conditioned NRVCM were labeled with Annexin V and tested for apoptosis by FACS. Finally, coculture supernatants were concentrated and used for ELISA and stimulation of NRVCM.

**Figure 2:**
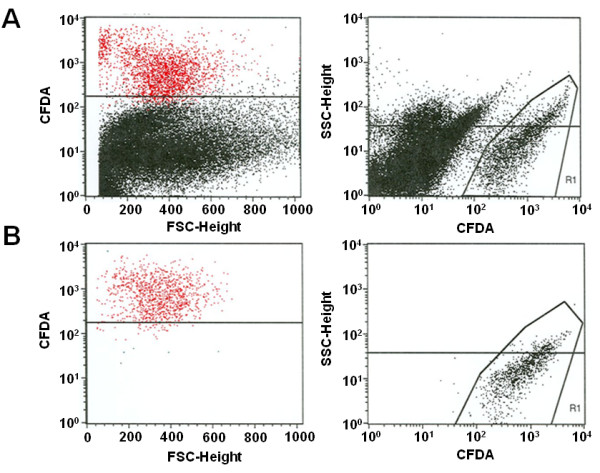
**illustrates the FACS analysis of CFDA labeled cocultured hematopoietic SC.** (**a**) Here we show the coculture prior to separation. After two consecutive cell sorting steps a highly purified population of hematopoietic SC with a purity >99.5% were generated and used for further experiments (**b**).

In order to elucidate potential molecular mechanisms of SC mediated cardiac regeneration and repair, we established a coculture system of CFDA labeled hematopoietic SC and NRVCM at a ratio of 1:4 per well (Figure 1). After 48 h of coculture, cells were separated by FACS (Figure 2A). Two consecutive cell-sorting steps enabled us to generate a highly purified (purity >99.5%) population of hematopoietic SC that were stimulated by NRVCM for 48 h (Figure 2B). These cells were subsequently used for further microarray analyses and compared to identically treated hematopoietic SC that were held in a monoculture for 48 h.

### Microarray analysis of hematopoietic SC after coculture with NRVCM

**Figure 3:**
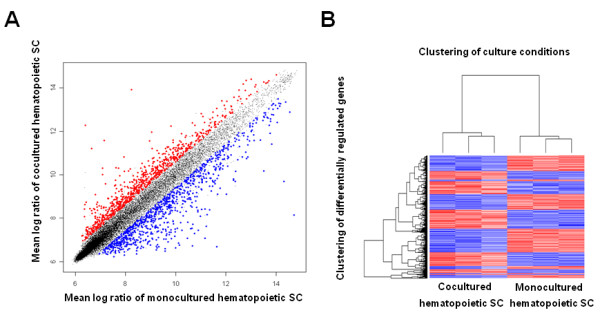
**displays the microarray analysis of cocultured hematopoietic SC.** (**a**) Scatter plots showing the log ratios of the means of differentially regulated transcripts in cocultured hematopoietic SC plotted against monocultured hematopoietic SC. Red dots represent upregulation, blue dots downregulation. (**b**) Heat map displaying 3014 differentially regulated transcripts. Red colour represents upregulation, blue colour downregulation.

We hypothesized that the direct interaction of hematopoietic SC with NRVCM may lead to an upregulation of cardioprotective genes in SC. Therefore we performed a genome-wide transcriptome microarray analysis of cocultured versus monocultured hematopoietic SC. We used RNA of three independent coculture and three monoculture experiments, respectively. Bayes analysis of the raw data revealed 3014 differentially regulated transcripts. Genes were considered differentially regulated, when up- or downregulation exceeded 50%. Regulation status in the scatter blot is colour coded. Upregulated genes appear red, downregulated genes blue and genes with no differential regulation are represented by black dots. 1500 were significantly (p < 0.05) and more than 1.5 fold upregulated (Figure 3A), whereas 1514 were significantly downregulated (less than 0.5 fold). Next, resulting data were normalized and displayed in heat maps, revealing highly reproducible results within groups and a low variance between microarray hybridizations (Figure 3B). We hypothesized that the close interaction of hematopoietic SC with NRVCM may lead to differential regulation of genes that promote survival of NRVCM via inhibition of apoptosis. Therefore, further processing of data generated from the microarray analysis focused on upregulated genes with secretable products that interfere with apoptotic pathways. We used two different databases (LOCATE, PANTHER) to identify candidate genes that met these criteria. Thereby, we were able to restrict our list to 8 genes. Specifically, CXCL 1, CCL 6, CCL 12, EGFL 7, FN 1, GJA 5, MIF and TIMP were shown to be not only significantly upregulated in our microarray analysis but also fulfilled our above mentioned prerequisites. A more detailed list is provided in table 1. The data discussed in this publication have been deposited in NCBI's Gene Expression Omnibus [18] and are accessible through GEO Series accession number GSE21098 (http://www.ncbi.nlm.nih.gov/geo/query/acc.cgi? token = vvyhramgioykypi&acc = GSE21098). Interestingly, we found no evidence for any transdifferentiation of hematopoietic SC towards a cardiac lineage. Consistently, early cardiac-specific transcripts like Nkx2-5, MEF2C, GATA4, markers of the primary heart field like Tbox5 and Hand1 or secondary heart field such as Isl1 and Fgf10, were not found to be differentially expressed, neither were later cardiac specific transcripts like alpha-MHC, Troponin-T, alpha-actinin, and Troponin-I. Flk-1, which is known to be a marker for multilineage mesoderm progenitor cells, was not differentially expressed as well. In addition, these findings confirm the high purity of our cell separation, resulting in no measurable contamination of hematopoietic SC with NRVCM RNA.

**Table 1:** Differentially regulated antiapoptotic genes in cocultured hematopoietic SC detected by microarray analyses

**Symbol**	**Gene-Name**	**Fold-Change**	***p*-Value**
**CCL6**	Chemokine (C-C motif) ligand 6	3.04	0.01
**CCL12**	Chemokine (C-C motif) ligand 12	4.65	0.029
**CXCL1**	Chemokine (C-X-C motif) ligand 1	4.42	0.049
**EGFL7**	EGF-like domain 7, transcript variant c	4.18	0.002
**FN1**	Fibronectin 1	5.16	0.017
**GJA5**	Gap Junction membrane channel protein alpha 5	1.57	0.001
**MIF**	Macrophage migration inhibitory factor	2.82	0.004
**TIMP**	Tissue inhibitor of metalloproteinase	1.44	0.004

Moreover, our number of differentially regulated genes in the microarray analysis was rather high. We have therefore added a list of genes that had an adjusted p-value of <0.05 and an upregulation of more than 1.5-fold. Next, we used DAVID Bioinformatics Ressources v6.7 to functionally annotate our transcripts via Gene Ontology [19,20]. We selected transcripts with the following Gene Ontology terms for further analysis: Apoptosis, Angiogenesis, Proliferation, Heart development, Immune response, Cell migration, Cell growth. These genes were classified according to their biological function. This list is added to the manuscript as Additional file 2: Table S1.

### Confirmation of microarray data by real-time PCR

**Figure 4:**
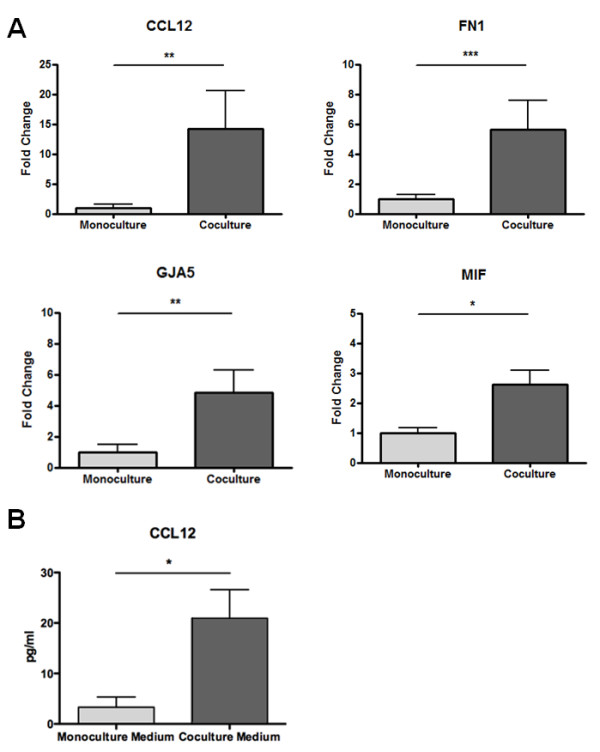
**demonstrates that coculture with NRVCM leads to overexpression of antiapoptotic factors in hematopoietic SC.** (**a**) CCL-12, FN-1, GJA-5 and MIF mRNAs are upregulated in cocultured hematopoietic SC (n = 8). Levels of significance *p < 0.05, ** *p* < 0.01, *** *p* < 0.001. (**b**) ELISA from concentrated cell culture supernatants show higher CCL-12 levels in coculture supernatants compared to monoculture supernatants (n = 3). Level of significance *p < 0.05.

We used quantitative Real-Time PCR to verify differential expression of selected genes in cocultured and monocultured hematopoietic SC. We primarily focused on genes with potentially secretable and antiapoptotic products for which an upregulation was predicted by our microarray data. Thereby we were able to show that coculture of hematopoietic SC with NRVCM for 48 h leads to an 14.8 fold upregulation of the chemokine (C-C motif) ligand 12 (CCL12) (*p* < 0.05), whereas expression of the gap junction membrane channel protein alpha 5 (GJA5, Connexin 40) was 4.5 fold induced (*p* < 0.05 versus control). Furthermore, real-time data revealed a significant 1.8 fold upregulation of the macrophage migration inhibitory factor and a 5.6-fold overexpression of Fibronectin in conditioned hematopoietic SC (*p* < 0.001) (Figure 4A). In contrast, we could not confirm significant differential expression of CXCL1, CCL 6, EGFL 7 or TIMP by real-time PCR, although the trend of their differential expression was consistent with the microarray data (p > 0.05).

### Antiapoptotic proteins are elevated in supernatants of cocultured hematopoietic SC

We initially hypothesized that cocultured hematopoietic SC express antiapoptotic proteins that may exert beneficial effects on NRVCM survival via a paracrine mechanism. We therefore collected and analyzed supernatants of several coculture experiments for increased levels of proteins identified in the microarray experiments. We measured the concentration of CCL12 in concentrated supernatants of three independent mono- and coculture experiments by ELISA. Thereby we were able to show that the CCL12 concentration is indeed significantly elevated in concentrated supernatants collected under coculture conditions when compared to monoculture experiments (21 pg/ml vs. 3,3 pg/ml; *p* < 0.05) (Figure 4B).

### Coculture with hematopoietic SC leads to inhibition of NRVCM apoptosis

**Figure 5:**
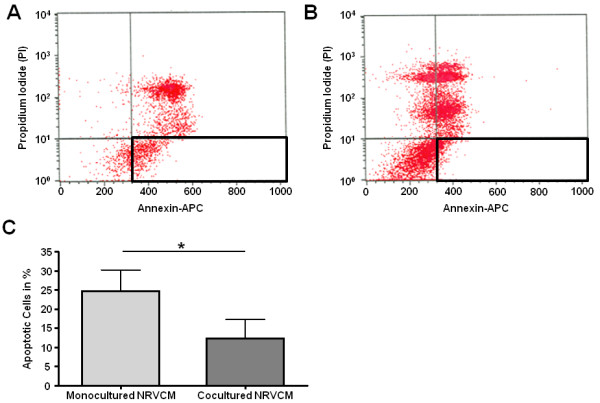
**confirms that coculture with hematopoietic SC inhibits apoptosis in NRVCM.** NRVCM were stained with Annexin-V-/Propidiumiodid and analyzed for apoptosis after coculture (**a**) or monoculture (**b**). Analysis of five experiments reveal significant less apoptotis in cocultured NRVCM (Annexin-V-positive, PI-negative cells) (**c**). All values are expressed as means +/− SEM. Level of significance: *p < 0.05.

Since our findings demonstrated that coculture of hematopoietic SC with NRVCM leads to an overexpression of secretable antiapoptotic proteins in SC, we were next asking whether these findings also translated into a reduced apoptosis rate in cocultured NRVCM. In order to address this question we repeated our coculture experiments with PI-/Annexin-V-staining. After 48 h of coculture and monoculture, we used APC-conjugated Annexin-V and PI to identify the fraction of apoptotic cells (Annexin V positive, PI negative) within the population of cocultured NRVCM (CFDA-/APC+/PI-) in comparison to identically stained NRVCM in monoculture. Indeed, coculture with hematopoietic SC cuts the number of apoptotic NRVCM by half when compared to monocultured NRVCM (12% vs. 24%; *p* < 0.05; n = 5) (Figure 5).

### Antiapoptotic effects are mediated via activation of the AKT/PKB pathway

**Figure 6:**
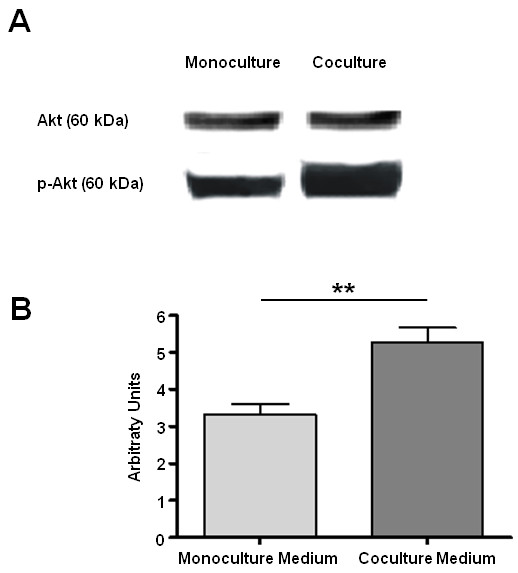
**reveals that antiapoptotic effects are mediated in an AKT dependent manner.** Western-Blot analysis shows that stimulation with concentrated coculture medium (n = 3) leads to higher AKT phosphorylation in NRVCM compared to stimulation with concentrated monoculture medium (n = 3) (**a**). Densitometric analysis reconfirms the significant difference in AKT phosphorylation (**b**). All values are expressed as means +/− SEM. Level of significance: *p < 0.05, **p < 0.01.

Finally, we sought to begin to investigate the underlying molecular mechanism that mediates antiapoptotic signals in cocultured NRVCM. In this context, it has been shown before that CCL 12, FN-1 and MIF can trigger phosphorylation of the antiapoptotic protein AKT via activation of PI3K [21-23]. We therefore tested the hypothesis that inhibition of apoptosis in cocultured NRVCM is related to an increased phosphorylation of AKT induced by factors secreted from hematopoietic SC in a paracrine manner. Hence, we used concentrated cell culture supernatants of several NRVCM/hematopoietic SC coculture and hematopoietic SC monoculture experiments to stimulate NRVCM that were held in a monoculture for 48 h. Densitometry of western-blots was utilized to compare the activation of selected signal transduction cascades. We found the anti-apoptotic AKT/PKB-pathway being significantly higher activated (ratio of Phospo-Akt473 to Akt) after stimulation with coculture medium compared to monoculture medium (5.2 vs. 3.2 arbitrary units, *p* < 0.01) (Figure 6). These findings suggest that survival of NRVCM that are held in a coculture with hematopoietic SC is improved by paracrine activation of the AKT/PKB signal transduction cascade.

## Discussion

Despite the fascinating idea of myocardial regeneration by undifferentiated precursor cells, the observed clinical effects of cellular cardiomyoplasty so far are rather modest and the underlying molecular mechanisms still remain unclear. One of the proposed mechanisms is the secretion of paracrine factors by SC that can modify cardioprotective signaling. The identification of these cytokines would be of high clinical relevance, since it may offer the possibility to establish new therapeutic options after myocardial infarction.

We thus hypothesized that a systematic analysis of the differential gene expression of hematopoietic SC after coculture with NRVCM may lead to the recognition of some of these factors. We here demonstrate that cell-cell interaction with NRVCM indeed results in an upregulation of secretable antiapoptotic proteins in hematopoietic SC which in turn improved NRVCM survival by paracrine pathways.

We used a genomic approach to identify genes overexpressed in hematopoietic SC after coculture with NRVCM. In the search of factors with therapeutic potential after myocardial infarction we restricted our analysis to genes that are not only significantly upregulated but also encoded for secreted or membrane-bound proteins. Moreover, we finally selected proteins that are also known to interfere with antiapoptotic signaling. Using such a systematic approach we were able to narrow down an initial list of 3014 differentially regulated genes to 8 genes. Biological validation of our microarray data by Real-Time PCR confirmed significant results for CCL-12, MIF, FN-1 and GJA-5, while there was only a strong trend towards upregulation for CXCL1, CCL 6, EGFL 7 and TIMP. Hence, our results demonstrate that coculture with NRVCM leads to a change in the secretome of hematopoietic SC.

Chemokine ligand 12 (CCL-12) is a small cytokine belonging to the C-C motif chemokine family. It is also known as monocyte chemotactic protein 5 (MCP-5) or as MCP-1 related chemokine. CCL-12 has been previously shown to be constitutively expressed in lymph nodes and thymus under basal conditions [24,25]. CCR2 is the receptor for CCL-12, which is not only expressed on monocytes and activated lymphocytes, but can also be found on cardiomyocytes [26]. Several experimental studies in rat neurons and murine lymphocytes indicated that stimulation of CCR2 can result in PI3K-dependent phosphorylation of AKT [27]. Thus, the increased phosphorylation of AKT observed in cocultured NRVCM may at least in part be explained by the paracrine action of CCL-12.

Another interesting candidate found upregulated in our experiments is the macrophage migration inhibitory factor (MIF). MIF is a pleiotropic cytokine which regulates the release of other pro-inflammatory cytokines and therefore is of great importance in mediating inflammatory responses [28]. MIF is known to be expressed and released upon stimulation from preformed storage pools by several cell types, including macrophages/monocytes, vascular smooth muscle cells and cardiomyocytes [29-31]. It has been shown that signal transduction of extracellular MIF involves a receptor tyrosine kinase (RTK)-like complex [32], promoting the activation of the PI3K/Akt pathway and cellular survival [22]. The biological relevance of this pathway has also been shown in the heart. Miller et al. demonstrated that MIF protects the rat heart from ischemia-reperfusion injury by stimulation of AMP-activated protein kinase (AMPK) in an autocrine manner [33]. Taken together, our results indicate that coculture of hematopoietic SC with NRVCM lead to an enhanced secretion of MIF by SC which may in turn protect NRVCM from apoptosis via phosphorylation of Akt in a paracrine manner.

Furthermore, it has become increasingly clear that interactions between extracellular matrix proteins and integrins not only mediate cell adhesion but also generate signals that play an important role in promoting cell survival [34]. Of note, Fibronectin (FN), one of the extracellular adhesive glycoproteins involved in these processes, was also found to be differentially regulated in our coculture experiments. FN is considered to provide survival signals for many cell types through its RGD motif that predominantly interacts with ß_1_-integrins, including α_3_β_1_ and α_5_β_1_ integrins [35]. The latter integrin was identified as an important regulator of apoptosis and is also expressed in cardiomyocytes [36-38]. Moreover, it has been repeatedly demonstrated that downstream signaling of α_5_β_1_ integrin involves a PI3K-dependent phosphorylation of AKT [23,39]. We therefore hypothesize that coculture-induced overexpression of FN in hematopoietic SC also contributes to the protection of NRVCM against proapoptotic stimuli by an integrin mediated activation of the PI3K/AKT pathway.

Finally, our results demonstrated that Gap junction protein-5 alpha (GJA-5) is significantly upregulated in hematopoietic SC after coculture with NRVCM. GJA-5, which is also known as Connexin 40, plays an important role in the formation of gap junctions that are clusters of intercellular channels consisting of a hexameric assembly of proteins. These gap junction channels can link neighboring cells and thereby provide the molecular framework of intercellular communication [40]. Moreover, it has been shown that in a variety of cells overexpression of Connexin 40 leads to increased resistance against several types of injury including calcium overload and oxidative stress. Surprisingly, the protective activity of connexin proteins was found to be independent of gap junction channel function [41]. Our finding of GJA-5 overexpression in conditioned SC may thus also contribute to improved intercellular connections between cocultured cells and/or increased resistance against cellular injury.

Thus, at least to our knowledge, we here demonstrate for the first time a systematic analysis of the differential gene expression in adult hematopoietic SC modified by a coculture with NRVCM. Our results show that a direct interaction of hematopoietic SC with NRVCM leads to an overexpression of antiapoptotic proteins in adult hematopoietic SC which may improve NRVCM survival in a paracrine manner. These data are in line with previous reports. The Dzau group showed that cell culture medium conditioned by hypoxic mesenchymal SC that were transduced with an Akt-1 overexpressing retrovirus reduced apoptosis and necrosis in isolated NRVCM [42]. As the putative underlying mechanism the same group presented data that revealed upregulation of potentially cytoprotective molecules such as VEGF, FGF, HGF or IGF in hypoxic and genetically modified mesenchymal SC [11]. Other groups have confirmed the paracrine effects of bone marrow derived SC on ischemic CM [43,44]. Uemura et al. demonstrated that hypoxia induced apoptosis of CM could be reduced by coculture with bone marrow derived mesenchymal SC. Since VEGF, bFGF, IGF and SDF-1 were found in the supernatant of mesenchymal SC culture, this group also argued for a paracrine mediated effect [45]. Similar results were also reported in a clinical setting. Korf-Klingebiel et al. used blood samples from 15 patients with acute myocardial infarction and isolated unselected nucleated bone marrow cells (BMSCs) and peripheral blood leukocytes (PBL). Cells were cultured to obtain conditioned supernatants. Cell culture medium of both cell types synergistically induced angiogenesis in a mouse aortic ring assay and protected rat CM from cell death induced by ischaemia followed by reperfusion [46].

How do our results fit into this context? We believe that our experimental approach is closer to the clinical setting, where bone marrow cells may directly interact with CM. While the above cited papers examined the generation of cytoprotective factors in various types of SC under monoculture conditions, we developed a coculture model that gave us the possibility to systematically analyze the differential gene expression in hematopoietic SC modified by direct interaction with NRVCM. We and others have shown that systemically infused or intramyocardially injected hematopoietic SC are to some extent retained in the ischemic myocardium [14,47]. Based on the results presented in this study we propose that a direct interaction with NRVCM induces the expression of antiapoptotic proteins in myocardially retained hematopoietic SC which in turn may contribute to the beneficial effects of cellular cardiomyoplasty. Of note, we also found several genes differentially regulated that play a role in other potentially cardioprotective pathways such as angiogenesis and immune response. Thus, it seems likely that in vivo additional mechanisms contribute to the effects of hematopoetic stem cells.

Some limitations of our work need further appreciation. For several reasons we preferred a genomic approach to systematically analyze the effects of coculture on adult hematopoietic SC. Compared to a proteomic approach a genomic approach is technically less demanding, more comprehensive and offers the possibility to detect new genes with previously unknown effects in SC therapy. On the other hand, a genomic approach can overlook important posttranscriptional events such as alternative splicing. Furthermore, it was beyond the scope of this study to analyze the therapeutic potentials of novel cytoprotective factors. Further studies will be needed to determine the therapeutic potentials of overexpressing factors such as CCL-12, MIF, FN or GJA-5. Finally, despite the high degree of purification of hematopoetic stem cells in our experimental setting, we cannot exclude that other cell types, e.g. macrophages, also contribute to the observed effects.

## Conclusion

In summary, we here present for the first time a comprehensive analysis of the gene expression program of hematopoietic SC after coculture with NRVCM. Thereby we were able to demonstrate that the direct interaction of hematopoietic SC with NRVCM leads to an upregulation of secretable and/or membrane bound antiapoptotic proteins which in turn improve NRVCM survival in a paracrine manner. Further studies will have to show whether the use of single cytoprotective factors can achieve similar results compared to cellular therapy after myocardial infarction.

## Competing interests

The authors declare that they have no competing interests.

## Authors’ contributions

MR participated in conception and design of the study, collected and assemblied study material or patients, analyzed and interpreted data and drafted the manuscript. ML and CK participated in conception and design of the study, collected and assemblied study material or patients, analyzed and interpreted data. RW and VE also helped in the analysis and interpretation of experimental data. JK participated in the Collection/Assembly of study material or patients. HK participated in the design of the study and helped to draft the manuscript. Finally NF also helped to conceive the study design, analyzed and interpreted data and drafted the manuscript. All authors read and approved the final manuscript.

## Funding

This work was supported by internal funds of the University of Heidelberg and Kiel.

## Supplementary Material

Additional file 1 Processing of conditioned SC, NRVCM and supernatants is described in the supplementary methods section. Click here for file

Additional file 2 Table S1.Additional differentially regulated genes in cocultured hematopoietic SC listed according to their potential protective biological function. Click here for file
