# Supplementary material for: Coculture with hematopoietic stem cells protects cardiomyocytes against apoptosis via paracrine activation of AKT
Source: J Transl Med. 2012 Jun 6;10:115. doi: 10.1186/1479-5876-10-115 (PMC3408384; doi:10.1186/1479-5876-10-115)
Supplement: Additional file 1 — Processing of conditioned SC, NRVCM and supernatants is described in the supplementary methods section. [file 1479-5876-10-115-S1.doc]

**RNA isolation, purification and analysis**

Total RNA was isolated from conditioned and control lin-/c-kit+ stem cells using the TRIzol reagent (Invitrogen, Germany), according to the manufacturer’s protocol. RNA was resuspended in DEPC–treated H_2_O (Sigma). RNA integrity and purity were electrophoretically verified by ethidium bromide staining and measurement of OD_260_/OD_280_ absorption ratios. Total RNA was purified from contaminating genomic DNA by a DNase I digestion (AMPD1, Sigma) using Qiagen´s RNeasy-Mini-Kit according to the manufacturer’s instructions. The quality of total RNA was checked by gel analysis using the total RNA Nano chip assay on an Agilent 2100 Bioanalyzer (Agilent Technologies GmbH, Berlin, Germany). Only samples with RNA index values greater than 8.5 were selected for expression profiling. RNA concentrations were determined using the NanoDrop spectrophotometer (NanoDrop Technologies, Wilmington, DE).

**ELISA assays**

In order to quantify the amount of differentially regulated antiapoptotic proteins in the concentrated supernatants of co- and monoculture experiments, an ELISA for CCL12 was carried out with DuoSet ELISA Development Kit for mouse CCL12 (R&D Systems) according to manufacturer’s instructions. Secreted protein levels were normalised to 1x10^6^ BMSCs.

**Detection of apoptotic cardiomyocytes**

For the detection and quantification of apoptotic cardiomyocytes mono- and coculture experiments were repeated as described above. After harvesting, cells were stained with propidium iodide (Sigma) and APC-conjugated Annexin V (SouthernBiotec) according to manufacturer’s instructions. More than 50,000 labeled cells were acquired and analyzed using a FACS-Vantage-SE flow cytometry system running CellQuest software (BD). In a first analysis step, NRVCM and lin-/c-kit+ stem cells were discriminated according to the CFDA signal and to their different forward-scatter and side-scatter signals. In a second step, viable, apoptotic and necrotic NRVCMs were distinguished according to their PI and Annexin-V signals. Data were statistically evaluated using the Mann-Whitney U test.

**Stimulation of NRVCMs with concentrated conditioned media and Immunoblotting**

Cardiac myocytes were seeded at a density of 2x10e6 cell per well. After 48 hours medium was removed, and the cells were washed once with 1x PBS and set on serum free media for another 24 hours. Cells were stimulated for 10 minutes with cell culture supernatants from co- and monoculture experiments, previously concentrated with Amicon Ultra-15 Centrifugal Filter Units (Millipore). We used media from three different and independent cocultures and two different and independent monocultures and stimulated two wells per condition. Immediately afterwards, NRVCMs were harvested and lysed in RIPA buffer containing 10 mmol/l Tris, 15 mmol/l EDTA pH 7.5, 1% NP 40 (v/v), 0,5 % Sodium deoxycholate (w/v), 0,1 % SDS (w/v) (all from Sigma), Protease Inhibitor Cocktail Tablets (Roche) and Phosphatase Inhibitor Cocktails 1 and 2 (Sigma). After a brief freeze-and-thaw cycle and centrifugation step, whole cell lysate was obtained. Total cell extracts were resolved by SDS-PAGE, transferred to Immobilon-FL Transfer Membrane (Millipore, USA) and immunoblotted with Akt and Phospho-Akt-Ser473 antibody. (Cell Signaling, #9272 and #4051) Proteins were visualized using Goat-anti-Mouse IR Dye 680, Goat-anti-Rabbit IR Dye I 800 CW secondary antibodies and an Odyssey Infrared Imager (all from LI-COR).
