# Supplementary material for: Coculture with hematopoietic stem cells protects cardiomyocytes against apoptosis via paracrine activation of AKT
Source: J Transl Med. 2012 Jun 6;10:115. doi: 10.1186/1479-5876-10-115 (PMC3408384; doi:10.1186/1479-5876-10-115)
Supplement: Additional file 2 — Table S1.Additional differentially regulated genes in cocultured hematopoietic SC listed according to their potential protective biological function. [file 1479-5876-10-115-S2.doc]

| **ID** | **Gene symbol** | **Gene Name** | **Gene Ontology** | **adjusted p-Value** | **Fold change** |
| --- | --- | --- | --- | --- | --- |
| NM_007413 | Adora2b | adenosine A2b receptor | Angiogenesis | 0,0089 | 2,55 |
| NM_007707 | Socs3 | suppressor of cytokine signaling 3 | Angiogenesis | 0,0018 | 5,96 |
| NM_008121 | Gja5 | gap junction membrane channel protein alpha 5 | Angiogenesis  Apoptosis | 0,0015 | 1,57 |
| NM_008155 | Gpi1 | glucose phosphate isomerase 1 | Angiogenesis | 0,0172 | 1,58 |
| NM_009382 | Thy1 | thymus cell antigen 1, theta | Angiogenesis | 0,0011 | 9,21 |
| NM_009932 | Col4a2 | collagen, type IV, alpha 2 | Angiogenesis | 0,0367 | 2,12 |
| NM_010941 | Nsdhl | NAD(P) dependent steroid dehydrogenase-like | Angiogenesis | 0,0475 | 1,72 |
| NM_015734 | Col5a1 | collagen, type V, alpha 1 | Angiogenesis | 0,0044 | 1,98 |
| NM_019389 | Cspg2 | versican | Angiogenesis | 0,0356 | 3,21 |
| NM_198725, NM_198724 | Egfl7 | EGF-like domain 7 | Angiogenesis  Apoptosis | 0,0019 | 4,18 |
| NM_176996 | Smo | predicted gene 4066; smoothened homolog (Drosophila) | Angiogenesis, Apoptosis, Cell Migration, Proliferation | 0,0038 | 1,65 |
| NM_008884 | Pml | promyelocytic leukemia | Angiogenesis, Apoptosis, Proliferation | 0,0095 | 1,71 |
| NM_010288 | Gja1 | gap junction protein, alpha 1 | Angiogenesis, Cell Migration, Cell growth | 0,0218 | 2,77 |
| NM_008361 | Il1b | interleukin 1 beta | Angiogenesis, Cell Migration, Immune response | 0,0014 | 5,36 |
| NM_011580 | Thbs1 | thrombospondin 1; similar to thrombospondin 1 | Angiogenesis, Immune response | 0,0414 | 2,48 |
| NM_008021 | Foxm1 | forkhead box M1; RIKEN cDNA 4933413G19 gene | Angiogenesis, Proliferation | 0,0112 | 1,93 |
| NM_007891 | E2f1 | E2F transcription factor 1 | Apoptosis | 0,0264 | 1,51 |
| NM_007931 | Endog | endonuclease G | Apoptosis | 0,0480 | 1,51 |
| NM_008842 | Pim1 | proviral integration site 1 | Apoptosis | 0,0066 | 3,27 |
| NM_009689 | Birc5 | baculoviral IAP repeat-containing 5 | Apoptosis | 0,0227 | 2,43 |
| NM_009742 | Bcl2a1a | B-cell leukemia/lymphoma 2 related protein A1a | Apoptosis | 0,0004 | 2,69 |
| NM_009811 | Casp6 | caspase 6 | Apoptosis | 0,0137 | 2,16 |
| NM_010774 | Mbd4 | methyl-CpG binding domain protein 4 | Apoptosis | 0,0020 | 1,55 |
| NM_011157 | Prg | serglycin | Apoptosis | 0,0181 | 1,71 |
| NM_011221 | Purb | purine rich element binding protein B | Apoptosis | 0,0421 | 1,70 |
| NM_011593 | Timp1 | tissue inhibitor of metalloproteinase 1 | Apoptosis | 0,0376 | 2,71 |
| NM_013787 | Skp2 | S-phase kinase-associated protein 2 (p45) | Apoptosis | 0,0245 | 1,52 |
| NM_013929 | Siva | SIVA1, apoptosis-inducing factor pseudogene; similar to CD27-binding (Siva) protein isoform 2; SIVA1, apoptosis-inducing factor | Apoptosis | 0,0085 | 1,64 |
| NM_019955 | Ripk3 | receptor-interacting serine-threonine kinase 3 | Apoptosis | 0,0041 | 1,70 |
| NM_023680 | Tnfrsf22 | tumor necrosis factor receptor superfamily, member 22 | Apoptosis | 0,0076 | 1,66 |
| NM_025374 | Glo1 | glyoxalase 1 | Apoptosis | 0,0232 | 2,03 |
| NM_026313 | 3300001P08Rik | RIKEN cDNA 3300001P08 gene | Apoptosis | 0,0142 | 1,81 |
| NM_028133 | Egln3 | EGL nine homolog 3 (C. elegans) | Apoptosis | 0,0136 | 4,35 |
| NM_028283 | Uaca | uveal autoantigen with coiled-coil domains and ankyrin repeats | Apoptosis | 0,0214 | 1,76 |
| NM_053272 | Dhcr24 | 24-dehydrocholesterol reductase | Apoptosis | 0,0273 | 1,90 |
| NM_134141 | Ciapin1 | cytokine induced apoptosis inhibitor 1 | Apoptosis | 0,0125 | 1,90 |
| NM_144899 | Tsrc1 | ADAMTS-like 4 | Apoptosis | 0,0094 | 1,89 |
| NM_153565 | Pcsk9 | proprotein convertase subtilisin/kexin type 9 | Apoptosis | 0,0012 | 3,57 |
| NM_176833 | Ppm1f | protein phosphatase 1F (PP2C domain containing) | Apoptosis | 0,0300 | 1,58 |
| NM_177733 | E2f2 | E2F transcription factor 2 | Apoptosis | 0,0310 | 1,76 |
| NM_010830 | Msh6 | mutS homolog 6 (E. coli) | Apoptosis, Immune response | 0,0075 | 1,67 |
| NM_007398 | Ada | adenosine deaminase | Apoptosis, Immune response, Proliferation | 0,0057 | 1,97 |
| NM_010253 | Gal | galanin | Apoptosis, Immune response, Proliferation | 0,0178 | 1,94 |
| NM_007669 | Cdkn1a | cyclin-dependent kinase inhibitor 1A (P21) | Apoptosis, Proliferation | 0,0100 | 3,02 |
| NM_007955 | Ptprv | protein tyrosine phosphatase, receptor type, V | Apoptosis, Proliferation | 0,0146 | 2,25 |
| NM_030693 | Atf5 | activating transcription factor 5 | Apoptosis, Proliferation | 0,0065 | 1,56 |
| NM_139117 | Csda | cold shock domain protein A | Apoptosis, Proliferation | 0,0477 | 1,58 |
| AK017632 | 5730441M17Rik | nucleoporin 85 | Cell Migration | 0,0125 | 1,80 |
| NM_010683 | Lamc1 | laminin, gamma 1 | Cell Migration | 0,0320 | 2,45 |
| NM_015779 | NE | elastase 2, neutrophil | Cell Migration, Immune response | 0,0080 | 1,51 |
| NM_010135 | Enah | enabled homolog (Drosophila) | Cell motion | 0,0449 | 1,56 |
| NM_053207 | Egln1 | EGL nine homolog 1 (C. elegans) | Heart development | 0,0050 | 1,82 |
| AK033202 | 8030459N02Rik | serum amyloid A-like 1 | Immune response | 0,0026 | 1,60 |
| NM_007800 | Ctsg | cathepsin G | Immune response | 0,0288 | 1,50 |
| NM_008176 | Cxcl1 | chemokine (C-X-C motif) ligand 1 | Immune response  Apoptosis | 0,0493 | 4,42 |
| NM_009139 | Ccl6 | chemokine (C-C motif) ligand 6 | Immune response  Apoptosis | 0,0101 | 3,04 |
| NM_009917 | Ccr5 | chemokine (C-C motif) receptor 5 | Immune response | 0,0167 | 3,19 |
| NM_010380 | H2-D1 | histocompatibility 2, D region; histocompatibility 2, D region locus 1 | Immune response | 0,0003 | 58,45 |
| NM_010381 | H2-Ea | histocompatibility 2, class II antigen A, alpha; histocompatibility 2, class II antigen E alpha | Immune response | 0,0324 | 23,29 |
| NM_010393 | H2-Q5 | histocompatibility 2, Q region locus 5 | Immune response | 0,0094 | 1,52 |
| NM_011331 | Ccl12 | chemokine (C-C motif) ligand 12; similar to monocyte chemoattractant protein-5 | Immune response  Apoptosis | 0,0288 | 4,65 |
| NM_011690 | Vars2 | valyl-tRNA synthetase | Immune response | 0,0252 | 1,67 |
| NM_018734 | Gbp4 | guanylate binding protein 3 | Immune response | 0,0266 | 1,95 |
| NM_019418 | Tnfsf14 | tumor necrosis factor (ligand) superfamily, member 14 | Immune response | 0,0198 | 2,63 |
| NM_026374 | Ilf2 | interleukin enhancer binding factor 2 | Immune response | 0,0123 | 1,67 |
| NM_133193 | Il1rl2 | interleukin 1 receptor-like 2 | Immune response | 0,0166 | 1,77 |
| NM_145545 | 9830147J24Rik | guanylate binding protein 6 | Immune response | 0,0020 | 2,20 |
| NM_207648 | H2-Q6 | histocompatibility 2, Q region locus 6 | Immune response | 0,0098 | 4,51 |
| NM_008489 | Lbp | lipopolysaccharide binding protein | Immune response, Cell Migration | 0,0049 | 2,04 |
| NM_010517 | Igfbp4 | insulin-like growth factor binding protein 4 | Immune response, Proliferation | 0,0013 | 3,12 |
| NM_010798 | Mif | macrophage migration inhibitory factor | Immune response, Proliferation, Apoptosis | 0,0387 | 2,38 |
| AK003784 | 1110018K11Rik | HOP homeobox | Proliferation | 0,0010 | 3,65 |
| AK046451 | B230386D16Rik | ankyrin repeat domain 11 | Proliferation | 0,0126 | 1,63 |
| AK084990 | Fanca | Fanconi anemia, complementation group A | Proliferation | 0,0382 | 1,59 |
| NM_007550 | Blm | Bloom syndrome homolog (human) | Proliferation | 0,0168 | 1,58 |
| NM_008048 | Igfbp7 | insulin-like growth factor binding protein 7 | Proliferation | 0,0376 | 3,75 |
| NM_008251 | Hmgn1 | high mobility group nucleosomal binding domain 1 | Proliferation | 0,0122 | 1,82 |
| NM_009829, AK077367 | Ccnd2 | cyclin D2 | Proliferation | 0,0051 | 3,75 |
| NM_010637 | Klf4 | Kruppel-like factor 4 (gut) | Proliferation | 0,0313 | 2,20 |
| NM_011407 | Slfn1 | schlafen 1 | Proliferation | 0,0423 | 2,88 |
| NM_020263 | Cacna2d2 | calcium channel, voltage-dependent, alpha 2/delta subunit 2; similar to Cacna2d2 protein | Proliferation | 0,0044 | 1,66 |
| NM_023065 | Ifi30 | interferon gamma inducible protein 30 | Proliferation | 0,0272 | 1,86 |
| NM_025378 | Ifitm3 | interferon induced transmembrane protein 3 | Proliferation | 0,0008 | 1,85 |
| NM_025866 | Cdca7 | cell division cycle associated 7 | Proliferation | 0,0188 | 1,74 |
| NM_058214 | Recql4 | RecQ protein-like 4 | Proliferation | 0,0111 | 1,62 |
| NM_133655 | Cd81 | CD81 antigen | Proliferation | 0,0079 | 1,55 |
| XM_130416 | 2810418N01Rik | GINS complex subunit 1 (Psf1 homolog) | Proliferation | 0,0180 | 1,91 |
| NM_010824 | Mpo | myeloperoxidase |  | 0,0116 | 2,61 |
